# 

**Published:** 1789

**Authors:** 


### C O N T E N T S.

Page

I. *SOME Obſervations on the medicinal Effects of the Lichen Iſlandicus and Arnica Montana. By* Alexander Crichton, *M. D.* - - - - - - - 229

II. *Obſervations on a Diſeaſe conſequent to tranſplanting Teeth. By Mr.* George Spence, *Dentiſt to His Majeſty.* - - - 243

III. *Remarks on febrile Contagion. By Mr.* James Lucas, *one of the Surgeons of the General Infirmary at Leeds, and Member of the Corporation of Surgeons in London.* - 260

IV. *Caſe of a fractured Scull unſucceſsfully treated. By Mr.* John Grimſton, *Surgeon at Ripon.* - - - - - - 277

V. *Some Account of the Tanjore Antidotes for the Bite of a mad Dog; and alſo for the Bite of venomous Serpents.* - - 283

VI. *Hints towards the Inveſtigation of the Nature, Cauſe, and Cure of the Rabies Canina: addreſſed to Dr.* Haygarth. *By* Thomas Percival, *M. D. Fellow of the Royal Societies of London and Edinburgh, of the Royal Medical Society at Paris, of the Royal Society of Agri-*

F f 2 *culture*

Page

*culture at Lyons, and of the Philosophical Society at Philadelphia,* &c. - - - - 295

VII. *Of the good Effects of a Decoction of the outer Shell of the Walnut in the Cure of Ulcers. By* J. Hunczoufky, *Professor of the Operations of Surgery, &c. in the Imperial and Royal Josephine Medico-Chirurgical Academy at Vienna.* - - - - - - 319

VIII. *An Account of a particular Change of Structure in the human Ovarium. By* Matthew Baillie, *M. D.—From the Philosophical Transactions of the Royal Society of London,* 322

CATALOGUE *of* BOOKS. - - 332

